# A phase II single arm study of Nivolumab with stereotactic Ablative radiation Therapy after induction chemotherapy in CHOlangiocarcinoma (NATCHO)

**DOI:** 10.1186/s12885-022-10373-1

**Published:** 2022-12-12

**Authors:** Charbel Elias, Youssef H. Zeidan, Youssef Bouferraa, Deborah Mukherji, Sally Temraz, Maya Charafeddine, Monita Al Darazi, Ali Shamseddine

**Affiliations:** 1 Department of Internal Medicine, Division of Hematology/Oncology, Naef K. Basile Cancer Institute- NKBCI, American University of Beirut Medical Center, Beirut, Lebanon; 2 Department of Radiation Oncology, American University of Beirut Medical Center, Beirut, Lebanon

**Keywords:** Nivolumab, Immunotherapy, PD-1 inhibitor, Cholangiocarcinoma, Biliary tract cancer, Radiotherapy, Chemotherapy

## Abstract

**Background:**

Intrahepatic cholangiocarcinoma (CCA) is amongst the most common primary liver tumors worldwide. CCA carries a bad prognosis prompting research to establish new treatment modalities other than surgery and the current chemotherapeutic regimens adopted. Hence, this trial explores a new therapeutic approach, to combine stereotactic body radiation therapy (SBRT) and immunotherapy (Nivolumab), and asses its clinical benefit and safety profile after induction chemotherapy in CCA.

**Methodology:**

This is a Phase II open-label, single-arm, multicenter study that investigates Nivolumab (PD-1 inhibitor) treatment at Day 1 followed by SBRT (30 Gy in 3 to 5 fractions) at Day 8, then monthly Nivolumab in 40 patients with non-resectable locally advanced, metastatic or recurrent intrahepatic or extrahepatic CCA. Eligible patients were those above 18 years of age with a pathologically and radiologically confirmed diagnosis of non-resectable locally advanced or metastatic or recurrent intrahepatic or extrahepatic CCA, following 4 cycles of cisplatin-based chemotherapy with an estimated life expectancy of more than 3 months, among other criteria. The primary endpoint is the progression free survival (PFS) rate at 8 months and disease control rate (DCR). The secondary endpoints are overall survival (OS), tumor response rate (TRR), duration of response, evaluation of biomarkers: CD3 + , CD4 + and CD8 + T cell infiltration, as well as any change in the PD-L1 expression through percutaneous core biopsy when compared with the baseline biopsy following 1 cycle of Nivolumab and SBRT.

**Discussion:**

SRBT alone showed promising results in the literature by both inducing the immune system locally and having abscopal effects on distant metastases. Moreover, given the prevalence of PD-L1 in solid tumors, targeting it or its receptor has become the mainstay of novel immunotherapeutic drugs use. A combination of both has never been explored in the scope of CCA and that is the aim of this study.

**Trial registration:**

ClinicalTrials.gov NCT04648319, April 20, 2018.

**Supplementary Information:**

The online version contains supplementary material available at 10.1186/s12885-022-10373-1.

## Background

Intrahepatic cholangiocarcinoma (CCA), which arises from the bile ducts [1], is amongst the most common types of liver tumors worldwide [2] making up around 3% of gastrointestinal cancers [3]. In the United States, CCA is the second most common primary hepatic malignancy with around 5,000 newly diagnosed cases per year [2]. With a 5-year survival of less than 10%, the prognosis is due to late stage presentation to clinic, high rate of metastasis and recurrence and the restricted amount of treatment modalities [4]. Although surgery persists as the only curative management plan, it is often not an option as only less than 20% of patients are candidates due to advanced stage at diagnosis. The remainder of the patients with advanced and unresectable CCA would undergo chemotherapy which presents with many limits [5–8]. With chemotherapy, the median OS for patients with an unresectable tumor is relatively low, ranging from 7 to 12 months. In addition, in surgical candidates, the recurrence rates reach up to 50%, and the post-resection survival rate at 5 years ranges between 8 to 44% [5, 7, 9–12]. In the absence of any treatment, the OS is 6 months regardless if the tumor is primary or recurrent [5, 9, 11–13].

Current chemotherapy regimens for patients with advanced CCA include gemcitabine, whether alone or in combination with platinum agents, or 5-fluorouracil [14]. Several studies have shown that the use of cisplatin and gemcitabine as a combination chemotherapy regimen has a significant survival advantage and can hence be used as a preferable treatment option for patients with advanced biliary cancer [14, 15]. Nevertheless, single or combination chemotherapy regimens have not been consistent in tumor shrinkage, prevention of recurrence, and increasing survival rate more than 8 to 15 months. As such, it is necessary to study and establish new treatment modalities in these patients. This protocol attempts to establish a new plan of treatment, composed of a potentially effective combination of radiotherapy and immunotherapy, or more specifically Stereotactic body radiation therapy (SBRT) and Nivolumab.

SBRT has been under the spotlight for a good while now. In a phase I trial 41 patients with inoperable cancer were recruited (10 intrahepatic CCAs and 31 hepatocellular carcinomas) to undergo SBRT (36 Gy over 6 fractions). Upon completion, the median OS for all patients was 13.4 months, survival at 1 year was 51%, and local control rates at 1 year were 65% [16]. Within the first 3 months, treatment related adverse events (AE) were restricted to grade 1–3 and no radiation-induced liver disease was noted. These favorable results led to more phase II and phase III trials studying the efficacy of 6 fraction SBRT. In another phase II trial, 46 patients with intrahepatic CCA were administered a high-dose conformal radiation therapy with concurrent hepatic artery floxuridine. Patients had a median OS of 13.3 months, which is improved versus historical control groups [17]. Consequently, SBRT is becoming more reliable as a management plan for patients with unresectable disease, as it seems to have promising clinical benefit and a good safety profile in these patients.

Moving on, the role of immunotherapy is being investigated in advanced biliary tract cancers (BTC). Durvalumab, an anti-PDL-1 agent, showed promising results in a phase I/II trial in comnation with tremelimumab in patients with HCC and BTC, with 42% of the patients in the BTC cohort achieving stable disease. Ongoing trials are evaluating the therapeutic efficacy of durvalumab in BTC as monotherapy or in combination with other checkpoint inhibitors, chemotherapy or local therapies [18]. Nivolumab is a monoclonal antibody that blocks PD-L1 and PD-L2 and their interactions with the PD-1 receptor. A single-group phase II study by Kim et al. (2020) administered nivolumab to 54 patients with BTC and showed that not only did it have a relatively good safety profile, but was also moderately effective and with durable response [19]. Another phase II trial by Klein et al. (2020) administered nivolumab and ipilimumab followed by nivolumab to 39 patients with advanced BTC. The trial concluded that there was significant positive outcomes that highlight the need to further investigate anti-PD1 treatment modalities [20].

It is only until recently that Nivolumab started gaining the spotlight in treating advanced CCA, as more clinical trials are currently emerging, combining nivolumab either with other immunotherapy medications or with chemotherapy or with other modalities [21]. Thus, Nivolumab has yet to be extensively and meaningfully studied in advanced BTC, despite the medication demonstrating clinical benefit in several different tumor types (i.e. melanoma, Hodgkin lymphoma, renal cell carcinoma, urothelial carcinoma, squamous cell cancer of the head and neck, and non-small cell lung cancer) and treatment settings [22–28]. Across the aforementioned tumor types, responses with nivolumab were typically observed early during treatment and appear durable in nature. In addition, studies have found that SBRT can produce a synergistic effect with immunotherapy, whereby it elicits changes in the tumor microenvironment, allowing for a better immune response. Dewan et at. demonstrate this in their study, where they showed that anti-cytotoxic T-lymphocyte associated protein 4 (CTLA4) antibody treated mice had an enhanced tumor response to fractionated radiotherapy (30 Gy in 5 sessions or 24 Gy in 3 sessions) at the primary site and at the site outside of the radiation field (abscopal effect) [29].

The current gold standard of therapy for advanced BTC is gemcitabine plus cisplatin as per the National Comprehensive Cancer Network (NCCN) guidelines (version 2021) [30]. The ABC-02 trial followed the same latter regimen [31]. The current trial population and that of ABC-02 share many similarities, which enables us to consider their population as a historical control group. No previous RCT data are available for the combination of high dose SBRT and Nivolumab in locally advanced CCA and there are no on-going trials regarding this topic. The progression free survival (PFS) is expected to reach 42% at 8 months in comparison to 23% in the ABC-02 study. Accordingly, the current trial efficacy and safety results will be compared to those of ABC-02 trial. In the ABC-02 randomized Phase II study, 86 patients with locally advanced or metastatic BTC were recruited to compare cisplatin plus gemcitabine with gemcitabine alone. The median PFS was 8 months in the cisplatin-gemcitabine group and 5 months in the gemcitabine-only group (*p*-value < 0.001). In addition, the rate of tumor control among patients in the cisplatin-gemcitabine group was significantly increased (81.4% versus 71.8%, *p*-value = 0.049). As such, the addition of cisplatin to gemcitabine was associated with a significant survival advantage without the addition of substantial toxicity [15].

In view of the above data, a Phase II clinical study is highly required to evaluate the PFS and the disease control rate (DCR) in patients with non-resectable locally advanced or metastatic or recurrent intrahepatic or extrahepatic CCA following nivolumab and SBRT. In our protocol, we assume that the addition of the immune checkpoint inhibitor, nivolumab, to SBRT will further improve outcomes including PFS and DCR [15].

## Methodology

### Objectives

This trial aims to assess the efficacy and safety of nivolumab with SBRT after induction chemotherapy in patients with CCA. The primary endpoint is the PFS at 8 months and DCR in patients with non-resectable locally advanced or metastatic or recurrent intrahepatic or extrahepatic CCA following Nivolumab/SBRT treatment.

The secondary endpoints are OS, tumor response rate (TRR), duration of response, evaluation of biomarkers: CD3 + , CD4 + and CD8 + T cell infiltration, changes in PD-L1 expression following 1 cycle of nivolumab and SBRT, AE and quality of life.

### Study design

This is a phase II open-label, single-arm, multicenter study investigating Nivolumab treatment at Day 1 followed by SBRT radiation treatment (30 Gy in 3 to 5 fractions) at Day 8, then monthly Nivolumab in patients with non-resectable locally advanced, metastatic or recurrent intrahepatic or extrahepatic CCA. One week following SBRT, PD-L1 and T cell infiltration changes will be assessed through percutaneous core biopsy as compared with the baseline biopsy, or from biopsy of the metastatic site in the case of extrahepatic CCA. In this study, 40 patients with non-resectable locally advanced or metastatic or recurrent intrahepatic or extrahepatic CCA patients will be enrolled in total.

This study has been prospectively registered in the registry of clinical trials of the United States of America (ClinicalTrials.gov Identifier: NCT04648319).

### Investigational medicinal product (IMP)

IMP description: Nivolumab (Opdivo®) for intravenous infusion, available as a solution of 10 mg/mL in single-dose vials. The active pharmaceutical ingredient in Nivolumab drug product is a human IgG4 monoclonal antibody, which binds to PD-1 receptor and blocks its interaction with PD-L1 and PD-L2.

#### Trial organization

This trial is principal-investigator initiated and sponsored by AUB (American University of Beirut). A total of four centers from three countries, Lebanon, Belgium and Luxembourg, are involved in the study. In Lebanon, the participating site is the American University of Beirut Medical Center (AUBMC). In Belgium, the two sites are Institut Jules Bordet and Cliniques Universitaires Saint-Luc. In Luxembourg, the participating site is Centre Hospitalier de Luxembourg.

#### Trial duration

There is no maximum duration of the treatment duration. Treatment will continue as patients are not progressing or not having any SAE that requires discontinuation of the treatment. The follow-up period is until death. The total period is 3 to 4 years.

### Statistics

For the efficacy endpoint, a land-mark analysis that might be more appropriate for immunotherapy was used to calculate the sample size. In the ABC2 study, the 12- month PFS, calculated from the start of chemotherapy, was 23% [15]. In this study, this would be equivalent to 8-month PFS as eligible patients will be recruited following 4 cycles of cisplatin-based chemotherapy administered over a period of 4 months. This study conservatively aims to increase the 12-month PFS, as defined by the ABC-02 study, to 42%. The sample size calculation comparing a single proportion to a known proportion (42%) yields a sample of 34 patients taking into consideration Type 1 error of 0.05 and power of 0.8. Adding that drop-out or treatment withholding due to patient decision or unacceptable AE occurrence can take place, an additional 15% will be added to yield a total sample size of 40 patients. PFS will be estimated using Kaplan–Meier methods. Median PFS and the PFS rate at 8 months and corresponding 95% CIs will be presented. PFS will be defined for all patients as the time between the date the patient is enrolled in the study and the date on which imaging reveals disease progression or the patient dies, whatever is earlier, and the last follow-up date, in case neither disease progression nor death has occurred. Patients without progression or death will be censored at the last assessment. PFS will be analyzed for all the patients. Exploratory variables will be analyzed according to their scale of measurement by using mean ± standard deviation or frequency distribution for numeric and categorical variables respectively. Frequency distribution for AEs and SAEs will be presented per cycle and per patient. Similarly, this will be done for the SAE of Grade 3 or above combined.

### Eligibility criteria

For patient inclusion, all of the following criteria must be met:

1. Signed and dated informed consent form.
2. Patients aged ≥ 18 years.
3. Pathologically (histologically or cytologically) and radiologically confirmed diagnosis of non-resectable locally advanced or metastatic or recurrent intrahepatic or extrahepatic CCA within 90 days of registration.
4. Patients who have SD or PR following 4 cycles of cisplatin-based chemotherapy.
5. ECOG performance score < 3.
6. An estimated life expectancy of more than 3 months.
7. Have adequate hematologic and biochemical function by meeting the following:
  - Total bilirubin acceptable level ≤ 1.5 × the institutional upper limit of normal (ULN) range;
  - Aspartate aminotransferase (AST) and alanine aminotransferase (ALT) acceptable levels up to 5 × ULN range;
  - Serum urea and serum creatinine acceptable levels up to 1.5 × ULN range;
  - Calculated glomerular filtration rate ≥ 45 mL/min according to the Chronic Kidney Disease Epidemiology Collaboration equation (or local institutional standard method).
8. Negative serum or urine pregnancy test at screening for women of childbearing potential who are sexually active.
9. Highly effective contraception for both males and females of child-bearing potential who are sexually active throughout the study and for at least 5 months and 7 months after the last nivolumab treatment administration, respectively.
10. Candidate for percutaneous biopsy as per tumor location evidenced by CT scan and interventional radiologist.

### Exclusion criteria

1. Patients who have progression following 4 cycles of cisplatin-based chemotherapy evidenced by CT scan as per RECIST 1.1.
2. Active brain metastases or leptomeningeal metastases.
3. Prior organ transplantation or allogenic stem-cell transplantation.
4. Known prior severe hypersensitivity to IMP or any component in its formulations, including known severe hypersensitivity reactions to monoclonal antibodies (NCI-CTCAE v4.03 Grade ≥ 3).
5. Active infection requiring systemic therapy within 28 days before the first dose of study treatment (e.g., urinary tract infection).
6. Known history of testing positive for the human immunodeficiency virus or known acquired immunodeficiency syndrome.
7. Evidence of liver cirrhosis.
8. Current use of immunosuppressive medication, except for the following:
  - Intranasal, inhaled, topical steroids, or local steroid injection (e.g., intra-articular injection);
  - Systemic corticosteroids at physiologic doses ≤ 10 mg/day of prednisone or equivalent;
  - Steroids as premedication for hypersensitivity reactions (e.g., CT scan premedication).
9. Active autoimmune diseases that might deteriorate upon receiving an immune-stimulatory agent.
10. Conditions such as vitiligo, psoriasis, diabetes type I, or hypo- or hyper-thyroid diseases not requiring immunosuppressive treatment are eligible.
11. Commonly excluded conditions include: Addison’s disease, thyroiditis/Hashimoto’s thyroiditis, systemic lupus erythematosus, Sjogren’s syndrome, scleroderma, myasthenia gravis, Goodpasture’s syndrome, and Grave’s disease.
12. Hepatic insufficiency manifesting as clinical jaundice, hepatic encephalopathy, and/or variceal bleed within 60 days prior to study entry.
13. Transmural myocardial infarction within 6 months of enrollment; provided that anti-platelets cannot be stopped to perform percutaneous biopsy.
14. Congestive heart failure (≥ New York Heart Association Classification Class II) requiring hospitalization within the last 6 months provided that anti-platelets cannot be stopped to perform percutaneous biopsy.
15. Serious cardiac arrhythmia requiring medical treatment provided that anti-platelets cannot be stopped to perform percutaneous biopsy.
16. Recent cerebral vascular accident/stroke within 6 months of enrollment provided that anti-platelets cannot be stopped to perform percutaneous biopsy.
17. End-stage renal disease requiring dialysis.
18. Other severe acute or chronic medical conditions including immune colitis, inflammatory bowel disease, immune pneumonitis, pulmonary fibrosis, or psychiatric conditions including recent (within the past year) or active suicidal ideation or behavior.
19. Treatment with an investigational agent within 28 days before the first dose of study treatment.
20. Prior treatment with any drug or antibody (anti-PD-1, anti-PD-L1, anti-PD-L2, anti-CD137, or anti-CTLA-4 antibody) targeting T cell co-stimulation or checkpoint pathways.
21. Patients suspected by the physician that he/she will not be compliant to the protocol conduct.
22. Pregnant women are excluded from this study; breastfeeding should be discontinued.
23. Patients participating in another clinical trial.
24. Patients not willing to sign the consent form.
25. Any psychiatric condition that would prohibit the understanding or rendering of informed consent.
26. Legal incapacity or limited legal capacity patients receiving other oncology specific medication not authorized in the protocol.

### Treatment plan (Fig. 1)

**Fig. 1:**
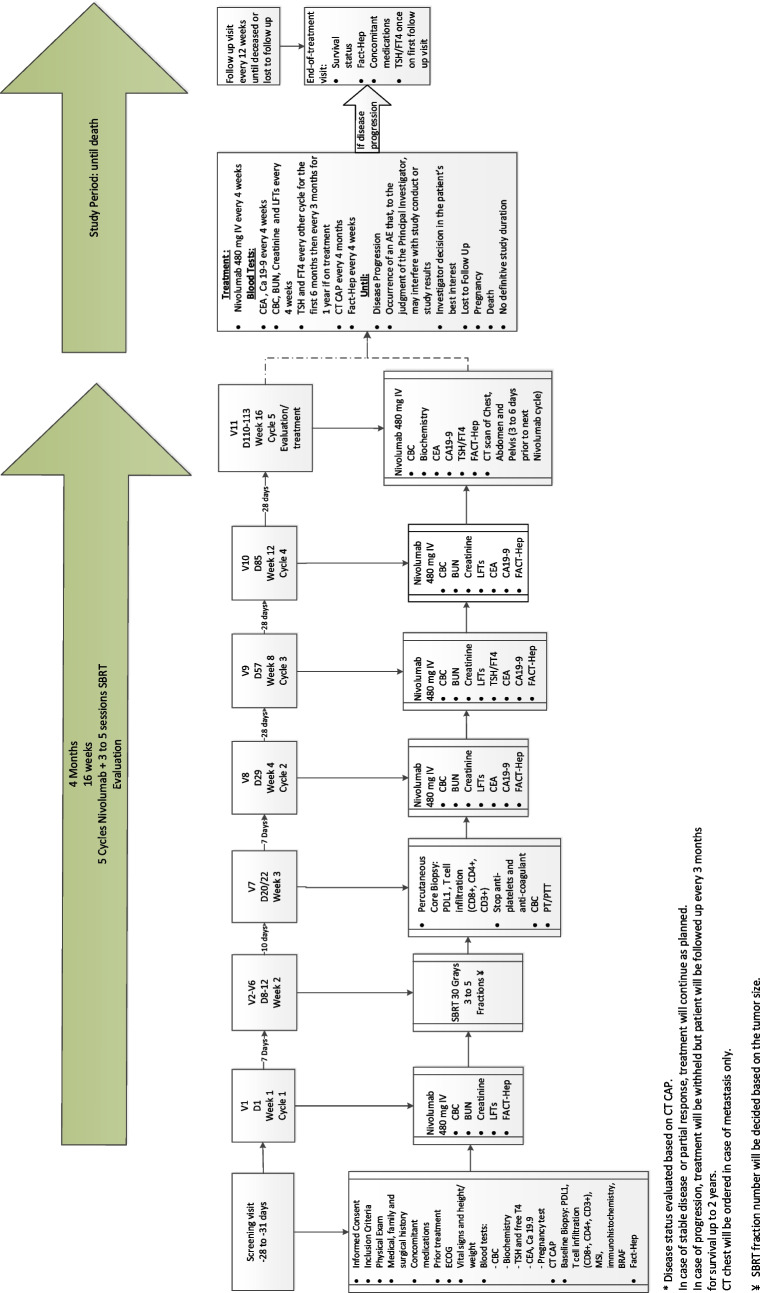
Treatment plan of the study, including all visits, cycles, labs and tests needed for monitoring

1. Nivolumab 480 mg IV over 60 min:
  Every 4 weeks;
  Until disease progression or unacceptable occurrence of AE
2. SBRT to 1–2 sites of disease starting around 7 days following the first Nivolumab dose:
  A total of 30 Gy in 3–5 fractions (depending on the target size and proximity to critical structures);
  The lesions treated with SBRT will be selected on individual basis using the following clinical overall guide:
    Symptomatic (pain or obstruction) lesions will be prioritized for SBRT
    Treatment safety and proximity to critical structures will be taken into consideration in choosing SBRT targets
  CT-based treatment planning will be performed for all participants. Whenever possible/deemed necessary, metal fiducials will be placed for intrahepatic targets. Image guided radiotherapy will be performed with cone beam CT scans taken prior to each fraction delivery.

### Treatment schedule

1. Week 1 (D1) ± 3 days: Nivolumab administration cycle 1.
2. Week 2 (D8-D12) ± 3 days: start of SBRT for 3–5 fractions.
3. Week 3 (D20 or D22) ± 3 days: percutaneous core biopsy 2 weeks after the end of SBRT.
4. Week 4 (D29) ± 3 days: Nivolumab administration cycle 2.
5. Week 8 (D57) ± 3 days: Nivolumab administration cycle 3.
6. Week 12 (D85) ± 3 days: Nivolumab administration cycle 4.
7. Week 16(D110-113) ± 3 days: Nivolumab administration cycle 5 and CT scan of the chest (to be ordered only in case of metastasis), abdomen and pelvis.

In case of disease progression: treatment will be withheld. Follow-up visits will be scheduled every 3 months. In case of stable disease or partial response: Nivolumab 480 mg administration every 4 weeks and disease evaluation by CT every 4 months of Nivolumab until disease progression.

### Outcomes

Tumor assessments will be done using CT scans of the abdomen, pelvis and chest.

PFS is defined for all patients as the time between the date the patient is enrolled in the study and the date on which imaging reveals disease progression or the patient dies, whatever is earlier, and the last follow-up date, in case neither disease progression nor death has occurred. Patients without progression or death will be censored at the last assessment regarding progression. PFS will be analyzed for all patients. OS is defined as the time from enrollment to death from any cause. TRR is the assessment of the tumor burden (TB) after a given treatment, duration of response is the time from response to progression/death. The following biomarkers will be evaluated: CD3 + , CD4 + and CD8 + T cell infiltration, and changes in PD-L1 expression following 1 cycle of Nivolumab and SBRT. AEs will be reported and graded according to NCI-CTCAEv5, within 24 h of knowledge of the event, to Bristol Myers Squibb (BMS) and the ethics committees, as per local rules and regulations. Quality of life will be assessed using the general questionnaire for the functional assessment of cancer therapy (FACT-Hep, English version 4 of 16 November 2007 or Arabic version 4 of 02 July 2018 or French version 4 of 19 February 2010).

## Discussion

Intrahepatic CCA carries an unfavorable prognosis with the 5-year survival rate at less than 10% [4]. This comes from the fact that CCA is usually diagnosed in advanced stages due to the nature of the disease. In addition, it is also due to the narrow availability of treatment options with surgical excision being the only curative route. However, few patients actually fit the criteria to undergo surgery and thus most of the patients are treated with palliative chemotherapy.

Regarding first-line chemotherapy, current regimens for patients with advanced CCA include gemcitabine with a platinum-based agent, typically cisplatin [30]. This combination has been proven to enhance the survival rate of patients [14, 15]. In the phase II ABC-01 trial, it was found that while both regimens gemcitabine and cisplatin/gemcitabine were beneficial in the treatment of advanced biliary cancers, cisplatin/gemcitabine proved to have better tumor control, time to progression and 6-months PFS than gemcitabine alone [32].

Nevertheless, the survival rate is still very limited and as such, second line regimens would then be used. In that regard, a retrospective study was done on 198 patients to document the different second line chemotherapy plans being used and their outcomes: a 5-fluorouracil-based therapy was used in 62% of patients whereas gemcitabine was employed in 18% of patients. The median time to treatment failure on second line chemotherapy was just 2.2 months, highlighting the limited efficacy of second line regimens and the need for new and more efficacious treatment plans [33]. To that end, the phase III ABC-06 trial established FOLFOX (folinic acid, fluorouracil, and oxaliplatin) as standard of care second line therapy for advanced biliary cancers versus ASC alone (active symptom control) which includes early identification and management of symptoms and complications that arise from tumor progression [34].

Moreover, new molecular targets such as the fibroblast growth factor receptor-2 (FGFR2) fusions and the Isocitrate dehydrogenase 1 (IDH-1) mutations are being studied in clinical trials [35]. In a phase II trial, the authors demonstrated the efficacy and benefits of pemigatinib, a FGFR1, FGFR2 and FGFR3 inhibitor, in treating advanced CCA with FGFR2 mutations [36]. Another phase II trial, BGJ398 exhibited significant clinical activity against refractory CCA with FGFR2 mutations [37]. As for IDH-1, a study sequenced and evaluated 94 specimens of CCA and found that 22% were positive for an IDH-1 mutation [38]. On top of that, a phase III study showed that ivosidenib, an IDH-1 inhibitor, is clinically beneficial in the treatment of IDH-1 mutant CCA [39]. Despite the availability of standard regimens and the promise of novel therapies, researchers continue to look for other parameters to explore as treatment for CCA, one of which being immunotherapy.

Anti-tumor T cells, also known as tumor infiltrating lymphocytes, are established prognostic markers in many solid tumors [40, 41] and specifically in CCA [42]. Moreover, a study completed by Park et al. (2015), found that out of 37 tumor tissue samples, 94% had PD-L1 expression, suggesting that new therapeutic modalities could potentially target PD-L1 and its PD-1 receptor through immunotherapy [43]. They also concluded that inhibiting these cell markers removes PD-1’s inhibitory function on T cells enhancing cancer cell destruction, and therefore has a promising role in treating CCA. Based on this, we chose to use Nivolumab as our investigational medicinal product (IMP); a human immunoglobulin G4 (IgG4) monoclonal antibody, which binds to the programmed death-1 (PD-1) receptor and blocks its interaction with PD-L1 and PD-L2. Engagement of PD-1 with the ligands PD-L1 and PD-L2, which are expressed in antigen presenting cells and may be expressed by tumors or other cells in the tumor microenvironment, results in inhibition of T cell proliferation and cytokine secretion. Nivolumab potentiates T cell responses, including anti-tumor responses, through blockade of PD-1 binding to PD-L1 and PD-L2 ligands. In a retrospective study that compared 75 patients who received a combination of PD-1 inhibitor with chemotherapy with 59 patients who had chemotherapy alone, it was found that PD-1 inhibitors plus chemotherapy improved PFS [44]. In a phase II study by Kim et al. (2020), it was found that Nivolumab showed moderate efficacy and durable response in patients with advanced refractory BTC [19]. Moreover, in a phase I study which employed two cohorts, Nivolumab monotherapy or in combination with cisplatin and gemicatabine, the authors found that Nivolumab exhibited promise in its clinical benefit and safety profile when used in unresectable or recurrent BTC [45]. In a phase II trial, Nivolumab was given in combination with cisplatin and gemcitabine where it was found to have potential clinical benefit and manageable safety in patients with advanced BTC [46]. Another similarly acting drug, pembrolizumab, a PD-1 inhibitor, was studied in two clinical trials: KEYNOTE-158 (NCT02628067), a phase II trial and KEYNOTE-028 (NCT02054806), a phase Ib trial. Both trials included patients with advanced incurable BTC after standard first line treatment, who received pembrolizumab, albeit while using different dosages. The reported overall response rate was 6% of patients in KEYNOTE-158 and 13% in those in KEYNOTE-028 [47]. Thus, pembrolizumab was found to be effective in 6–13% of patients with advanced BTC [48]. Pembrolizumab is further being studied in a phase III randomized clinical trial (KEYNOTE-966, NCT04003636), where it is added to gemcitabine and cisplatin in the experimental arm and is being compared to the control arm, which consists of gemcitabine, cisplatin, and placebo.

Another modality to consider is radiation therapy, which induces an immune response via several mechanisms culminating in immunogenic cell death. Radiation therapy, when given in high doses (such as in SBRT), has been shown to promote tumor cell lysis in the local targeted area. It also enhances the release of tumor-associated antigens (TAA) which can be taken up by professional antigen presenting cells (APC) and are then activated by proinflammatory cytokines [49, 50]. This signals the APC to migrate the lymph nodes near the tumor sites and activate CD8 + cytotoxic T cells [51]. In this way, through the use of TAA, radiation therapy can activate the immune response, specifically T cells, against tumor cells. In addition, SBRT carries the abscopal effect, which refers to the ability of SBRT to target distant sites beyond the local target area though the increase in circulation of pro-inflammatory cytokines released from both the immune and tumor cells [50, 52]. Furthremore, in a phase II study, 83 patients with unresectable tumors (either hepatocellular carcinoma or intrahepatic CCA) underwent high-dose hypofractionated proton therapy which was found to exhibit high rates of local control and survival for both hepatocellular carcinoma or intrahepatic CCA [53].

As for combining immunotherapy with SBRT, a study by Park et al. (2015) showed that clinically radiotherapy and anti PD-1 targeted therapy led to a near-complete regression of the primary tumor. They also reported a 66% regression in distant tumors, mainly in preclinical melanoma and renal cell carcinoma models, via abscopal responses, which are defined as the regression of metastatic cancer at a distance from the irradiated site following local radiotherapy [54]. Similarly, Postow et al. (2012) reported that palliative radiotherapy (28.5 Gy in 3 fractions delivered over 7 days) given concurrently with maintenance ipilimumab treatment in a patient with melanoma caused regression of the targeted lesion as well as marked abscopal effects [55].

**Fig. 2:**
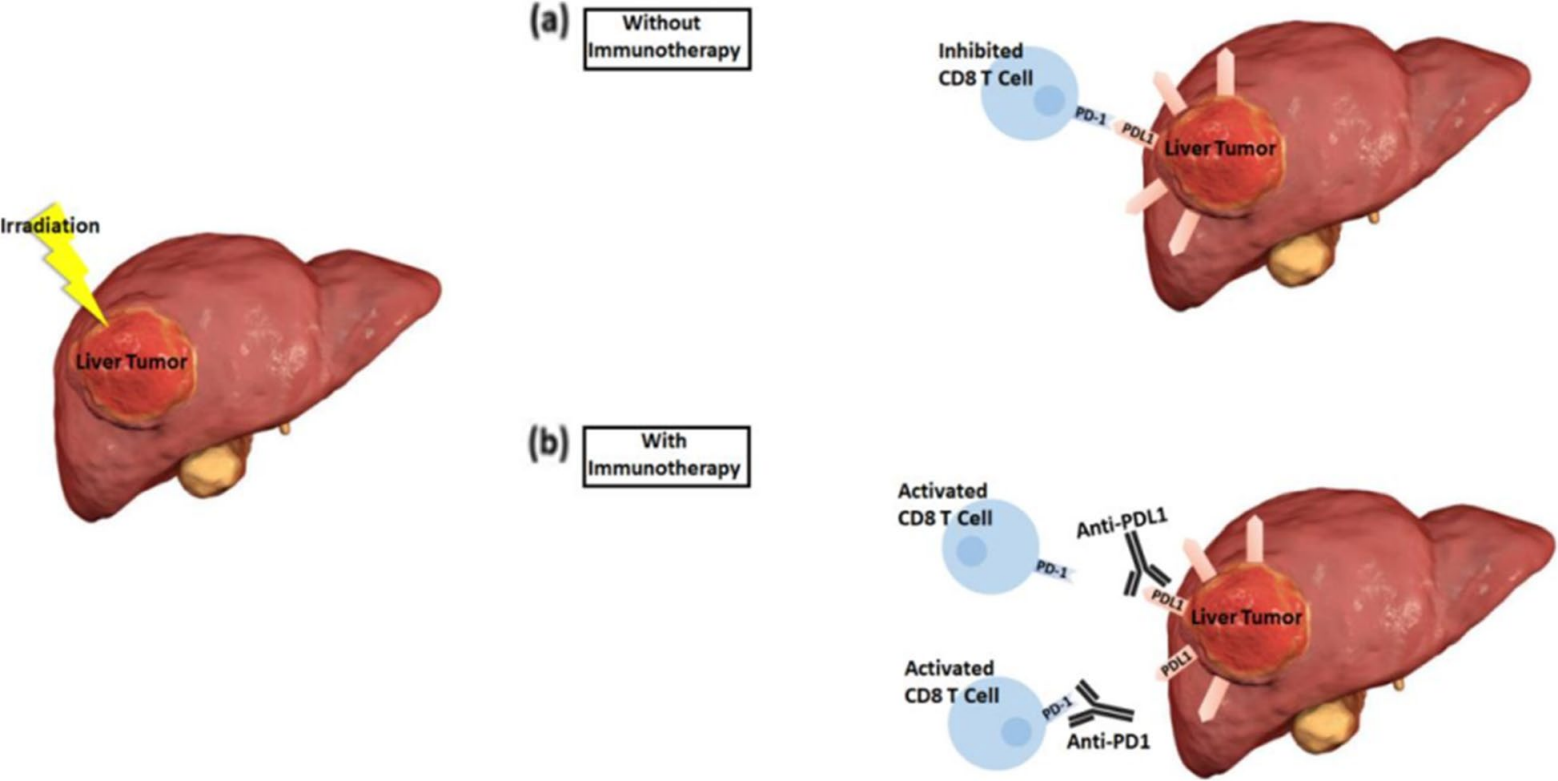
Schema of synergistic effect of SBRT and immunotherapy (Adapted from Kreidieh et al. [57])

The rationale behind combining immunotherapy with radiation therapy was also highlighted in a study by Deng et al. (2014). The authors discuss how an increase in PDL-1 is one of the causes of tumor relapse post-radiation. They went on to combine anti-PD-L1 antibody therapy with irradiation in the treatment of two allograft tumor models, TUBO breast cancer and MC38 colon cancer. They reported on the synergism between the two modalities in both tumor models versus minimal regression in the tumors after administering radiation alone. In fact, the combination also enhanced antitumor activity, and successfully targeted distant tumor sites via abscopal effect [56] (Fig. 2, adapted from Kreidieh et al. [57]). Therefore, the authors concluded that there is synergism between PD-1 blockade and radiation therapy [56]. Thus, while this combination proved effective in breast and colon cancers and while SBRT alone shows promise as an effective local therapy in patients with CCA [58], the combination of SBRT and PD-L1 or PD-1 inhibition has not been tested before in CCA. Based on the above, there is a molecular justification for the synergistic effect of radiation and PD-L1 inhibition that may apply to the combination of radiation and PD-1 inhibition as well.

There is a promising clinical role in using the combination of radiotherapy with targeted immunotherapy in CCA, based on the emerging data on immunotherapy in tumors along with the discoveries in radiation therapy [59]. The current protocol explores the clinical efficacy and safety profile of combining nivolumab with SBRT in patients with non-resectable locally advanced or metastatic or recurrent intrahepatic or extrahepatic CCA. Based on the evidence found in the literature highlighted above, we expect that Nivolumab in combination with SBRT to improve PFS and OS in these patients.

## Supplementary Information

**Additional file 1.**

## Data Availability

Data that support the findings of this study are stored in a secured server, in a manner that the data is not publicly available but restricted to the acknowledged study personnel as per study protocol. The research finding results will be available as abstracts published in journals, the NCT clinical trial website (https://www.clinicaltrials.gov/), and the European clinical trial website (https://eudract.ema.europa.eu/). Data processing, from data collection to database lock, will be carried out in accordance with Good Clinical Practice. The database structure, data entry manual, coding rules, and computerized validation are defined in a Data Management Plan. The database and data entry screens will be created in software specifically designed for clinical data management in compliance with ICH-E6 requirements. All eCRFs received in the Data Management Unit will be tracked by the Data Manager. The consistency of data will be checked by computerized programs and related queries will be generated for resolution by the Investigator. The database will then be updated accordingly. At the end database lock will be performed by the assigned data management team who will, consequently, be responsible for transferring the cleaned data into a statistical software for analysis. To access raw data, a request should be sent to the PI, Dr. Ali Shamseddine, via e-mail as04@aub.edu.lb, stating the reason for requesting the data and the list of variables needed. The PI will revise the request and grant permission accordingly. All data will be de-identified and encrypted.

## Abbreviations

CCA: Cholangiocarcinoma
OS: Overall survival
SBRT: Stereotactic body radiation therapy
AE: Adverse event
BTC: Biliary tract cancer
NCCN: National Comprehensive Cancer Network
PFS: Progression free survival
DCR: Disease control rate
TRR: Tumor response rate
TB: Tumor burden
AUB: American University of Beirut
AUBMC: American University of Beirut Medical Center
BMS: Bristol Myers Squibb
TAA: Tumor-associated antigens
APC: Professional antigen presenting cells
